# A decade of neonatal sepsis in Stockholm, Sweden: Gram-positive pathogens were four times as common as Gram-negatives

**DOI:** 10.1007/s10096-024-04809-8

**Published:** 2024-03-22

**Authors:** Frida Oldendorff, Viveka Nordberg, Christian G Giske, Lars Navér

**Affiliations:** 1 Astrid Lindgren Children’s Hospital, Karolinska University Hospital, Stockholm, Sweden; 2 Department of Neonatology, Astrid Lindgren Children’s Hospital, Karolinska University Hospital, Stockholm, Sweden; 3 Department of Clinical Science Intervention and Technology (CLINTEC), Division of Pediatrics, Karolinska Institutet, Stockholm, Sweden; 4 Department of Laboratory Medicine, Division of Clinical Microbiology, Karolinska Institutet, Stockholm, Sweden; 5 Clinical Microbiology, Karolinska University Hospital, Stockholm, Sweden

**Keywords:** Neonatal, Gram-positive, Sepsis, Group B streptococcus, *Staphylococcus aureus*, Coagulase-negative staphylococci

## Abstract

**Purpose:**

To assess Gram-positive bacterial (GPB) bloodstream infection (BSI) in neonates, covering incidence, morbidity, mortality, antimicrobial resistance patterns and biomarkers in Region Stockholm, Sweden between 2006 and 2016.

**Methods:**

A population-based retrospective epidemiological study including infants with GPB-BSI, admitted to the neonatal units at Karolinska University Hospital (KUH). Data were collected from patient records, the Swedish Neonatal Quality Register, the microbiological laboratory at KUH and the Swedish Public Health Agency.

**Results:**

We identified 357 infants with GPB-BSI, representing an incidence of 1.47/1000 live births (LB). Group B streptococcus (GBS) was the most common pathogen causing BSI in full-term infants and early-onset sepsis (EOS) (0.20/1000 LB), while coagulase-negative staphylococci (CoNS) were predominant in infants born very preterm and in late-onset sepsis (LOS) (0.79/1000 LB). There were no fatal GBS BSI cases, but 10.2% developed meningitis. The GPB case fatality rate was 9.5% and the sepsis fatality rate 2.8%. In GPB-BSI, 1/10 did not have an elevated C-reactive protein level. *Staphylococcus aureus* (*S. aureus*) BSI increased during the study period, but no methicillin or vancomycin resistant strains were found. The antimicrobial resistance (AMR) rate was highest in CoNS isolates.

**Conclusion:**

GPB-BSI was four times more common than Gram-negative BSI in neonates but resulted in lower mortality rate. GBS was the most common pathogen in full-term infants and in EOS. CoNS was the most common pathogen in LOS and infants born very preterm, and the AMR rate was high in these isolates. The increasing trend of *S. aureus* BSI indicates a need of further investigation.

**Supplementary Information:**

The online version contains supplementary material available at 10.1007/s10096-024-04809-8.

## Introduction

Neonatal sepsis globally affects around 4/1000 live born (LB) infants and is associated with a high mortality and morbidity rate, longer hospital stays and increased healthcare costs [1, 2]. The clinical presentation of neonatal sepsis varies depending on time of onset, gestational age, and causative pathogen [1, 2]. A broad spectrum of pathogens (i.e., bacteria, viruses and fungi) can cause neonatal sepsis [1]. Signs can initially be few and subtle like temperature instability, feeding difficulties, change in skin color, tachy- or bradycardia, tachypnea, apnea, grunting, irritability, or lethargy [1]. A positive culture from blood or cerebrospinal fluid (CSF) is a strong indicator of sepsis [1]. However,certain pathogens, like coagulase-negative staphylococci (CoNS), can be contaminants and cause falsely positive cultures [3]. Polymerase Chain reaction (PCR) is a sensitive diagnostic method of bacterial infections, which require smaller samples than blood/CSF cultures. Nonetheless, the high sensitivity of PCR increases the risk of false positive samples [3, 4]. Biomarkers like C-reactive protein (CRP) and platelet (PLT) count may be unreliable for neonates [5]. Further, cytokines, e.g., interleukin-6 (IL-6) has been suggested as an indicator of neonatal sepsis [6]. However, both CRP and IL-6 could be difficult to use as they require various cut-off levels at different time-points after birth [7]. Despite several attempts, there is no consensus definition of neonatal sepsis [2, 8].

The likelihood of a certain bacteria being the pathogen in neonatal sepsis varies depending on time of symptom onset. In early-onset sepsis (EOS), often defined as start of infection symptoms < 72 h of age, the infection is vertical, with pathogens often being group B streptococci (GBS) and *Escherichia coli* (EC), the former being a GPB [3–6]. The risk of EOS increases with the occurrence of chorioamnionitis, prolonged rupture of membranes (i.e., > 18 h before birth) and maternal GBS colonization [9]. In late-onset sepsis (LOS), symptoms occur at > 72 h of age, the infection is usually hospital-acquired and caused by Gram-positive bacteria (GPB), such as CoNS and *Staphylococcus aureus* [10–12]. Low birth weight and gestational age increase the risk of LOS [10, 12].

Prematurity is an established risk factor for neonatal infections [1]. This has resulted in an excessive use of antibiotics in neonatal units, which is sometimes crucial for the infant’s survival. For example, in the US, almost 90% of all extremely low birth weight neonates (i.e., birth weight < 1000 g) receive antibiotics during their first days in life [13]. Likewise, a higher gestational age has been shown to be a protective factor against morbidity and mortality in neonatal sepsis [14]. A recent point prevalence study, The Worldwide Antibiotic Resistance and Prescribing in European Children (ARPEC), examined antibiotic prescription in 240 neonatal intensive care units (NICUs) worldwide and found that 35% of all neonates received antibiotics [15]. Further, the study showed that the most prescribed antibiotics for nosocomial infections was vancomycin in several regions (including Europe), which has raised concerns, since it is classified as a ”watch” antibiotic in the WHO AWaRe classification [15, 16].

Simultaneously, an estimated 214,000 neonatal deaths from sepsis each year are caused by antimicrobial resistance (AMR) [17], and AMR in neonatal sepsis is increasing rapidly [18]. It has been estimated for late preterm and full-term neonates that around 50–100 infants are given antibiotics for every true EOS case in high income countries [19, 20]. This becomes further problematic as an association between antibiotic use in early life, dysbiosis of the neonatal intestinal microbiota and an increased risk of adverse short term- (e.g. NEC and LOS) and long term outcomes (e.g. asthma, inflammatory bowel disease and allergy) has been described [1, 21–24]. Further, there is a correlation between neonatal antibiotic exposure and an increase in antimicrobial resistance in the intestinal microbiome [25].

It is crucial for every neonatal unit to know the current spectrum of bacterial pathogens, as well as their AMR pattern and impact of neonatal sepsis to provide a safe but effective antibiotic stewardship. Currently, this information is insufficient and treatment decisions risk of being based on outdated knowledge. The aim of this study was to describe incidence, morbidity, mortality, AMR patterns, and biomarkers of GPB sepsis in neonates during an 11-years period in Region Stockholm, Sweden. The overarching goal was to increase the knowledge support for risk assessment, diagnosis, and treatment of Gram-positive infections in newborns. This study complements a previous study of sepsis caused by Gram-negative bacteria (GNB) in Region Stockholm over the same 11-year period (2006–2016) [14].

## Materials and methods

### Study design

This was a retrospective epidemiological study over 11 years (1 January 2006- 31 December 2016).

### Setting

Region Stockholm had 2,200,000 inhabitants in 2016. There were six delivery units and four hospitals that provide neonatal care. During March 2014-May 2016, a seventh delivery unit with a small level 1–2 neonatal unit, BB Sophia, operated. There are four neonatal intensive care units (NICUs), one at the South General Hospital and three at Karolinska University Hospital (i.e., Karolinska Solna, Karolinska Huddinge and Karolinska Danderyd). The three NICUs at Karolinska University Hospital are in different geographical areas in Stockholm and divided into one level 2- unit (i.e., for neonates born ≥ 32 gestational weeks (GW), whom need a high level of medical care) and two level 3-units (i.e., for all neonates who need the highest level of medical support for more than 48 h).

### Study population

The study population consisted of infants admitted to the three NICUs at Karolinska University Hospital, the NICU at the South General Hospital and the NICU at BB Sophia, who had at least one positive blood or cerebrospinal fluid culture with growth of a GPB within 28 days of life or until 4 weeks after they reached full-term. All positive blood cultures with growth of *Staphylococcus aureus* (*S. aureus*) and GBS were considered sepsis cases. Blood cultures that were simultaneously positive for one or more GPB were classified as a mixed infection and reported in a separate group. CoNS-positive blood cultures were divided into two subgroups: sepsis and probable contamination. For the definition of sepsis, the following criteria should be met: (1) a positive blood culture (2) clinical symptoms of sepsis (i.e., at least two of the following symptoms: general malaise, fatigue, poor skin color, tachypnea, tachycardia, or bradycardia) and (3) intravenous antibiotic treatment for at least 5 days after a positive blood culture or until death, if death occurs within 5 days after a positive blood culture. Cases with probable contamination was defined as failure to meet the stated sepsis criteria and were excluded from further analysis. Further, cultures with growth of *Bacillus cereus* (*n* = 2) and *Actinomyces naeslundii* (*n* = 1) were considered contaminants and excluded from the study. In addition, blood cultures that were simultaneously positive for fungi or GNB were excluded. Sepsis fatality rate (SFR) was defined as death within 5 days after first GPB positive blood culture. Meningitis was defined as the presence of a GPB growth in cerebrospinal fluid. Septic shock was defined as sepsis caused by a GPB, where the child was given extra fluid and/or an inotropic drug according to guidelines for treatment of a circulatory shock.

### Data collection

Both GPB and GNB isolates were identified. Data were collected from patient records from Stockholm’s five NICUs, the Swedish Neonatal Quality Register (SNQ), the microbiological laboratory at Karolinska University Hospital and from the Public Health Agency of Sweden. Data concerning neonatal sepsis with GNB is previously reported [14]. Due to lack of access to medical records, isolates from the South General Hospital and BB Sophia were thereafter excluded. Data from remaining three NICUs was retrieved from the electronic medical record system TakeCare and pseudonymized according to the General Data Protection Regulation.

### Statistical analyses

This was a retrospective descriptive study with varying length of hospital stay and time between onset of symptoms and discharge. Data on all live born infants in the study region, during the study period was collected from Statistics Sweden, (www.scb.se).

### Microbiological species identification and antimicrobial susceptibility testing

Species identification had been carried out using Matrix-Assisted Laser Desorption/Ionization Time Of Flight Mass Spectrometry (MALDI-TOF MS), and antimicrobial susceptibility testing using the disk diffusion method according to EUCAST, (www.eucast.org), except for vancomycin resistance which was investigated with the gradient test method (ETEST, bioMérieux, Askim, Sweden). In our region CoNS-isolates are not routinely subjected to identification at the species level and were therefore reported as a group in this study. All microbiological analyses were conducted at the clinical microbiology laboratory at Karolinska University Hospital.

## Results

### Incidence

**Fig. 1:**
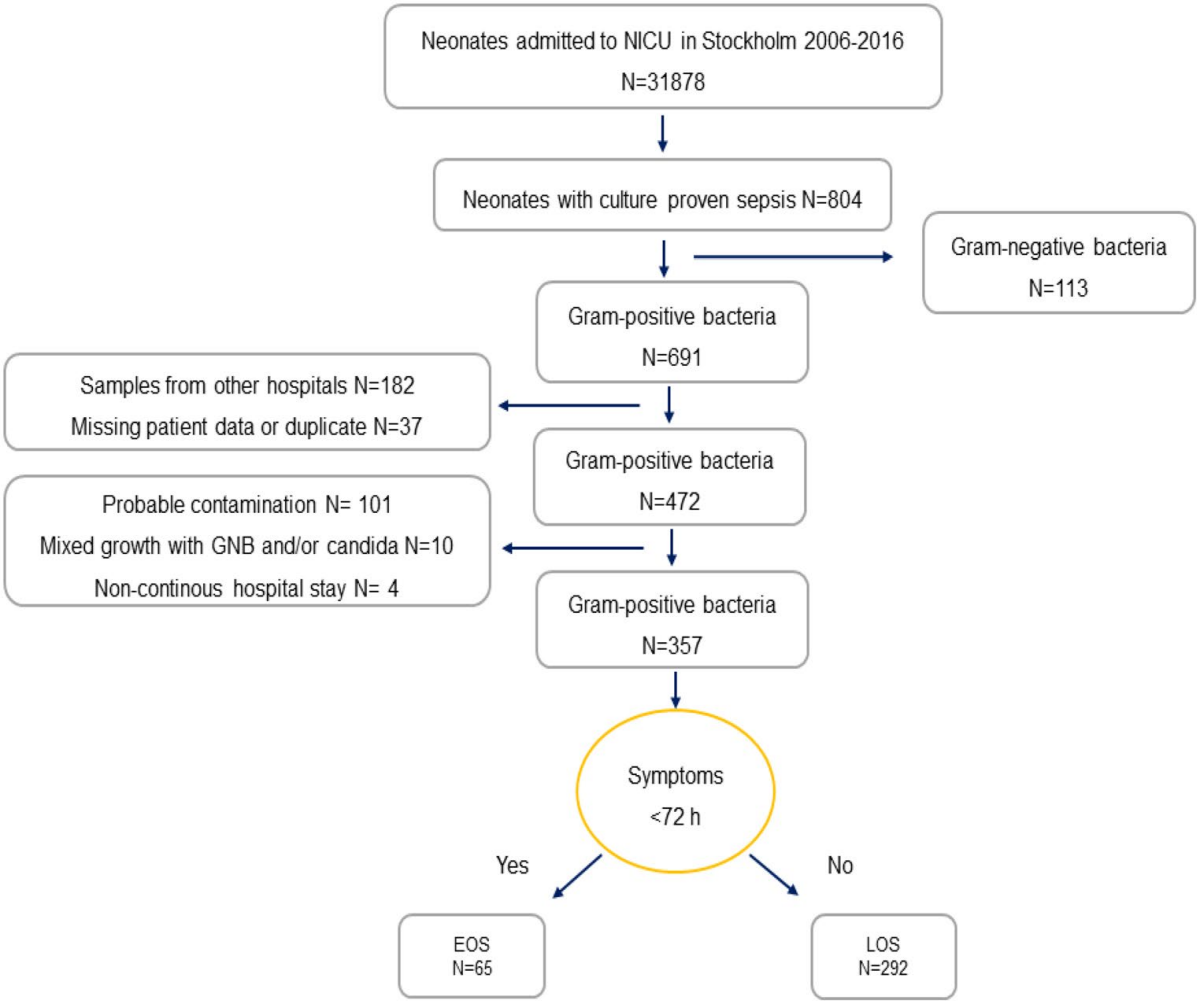
Flow chart over the inclusion process and categorization into EOS and LOS. *NICU* neonatal intensive care unit, *EOS* early-onset sepsis, *LOS* late-onset sepsis

During the study period a total of 316,070 infants were born in Region Stockholm, of which 31,878 were admitted to a NICU; at Karolinska Solna (*n* = 5,928), Karolinska Huddinge (*n* = 6,904), Karolinska Danderyd (*n* = 10,418) and the South General Hospital (*n* = 8,728). In this group, 804 cases of culture proven sepsis were found, equivalent to an incidence of 2.5/1000 live born (LB), of which 691 cultures were GPB (85.9%), see Fig. 1. After excluding samples from which further information could not be gathered; samples from South General Hospital and other hospitals outside the region (*n* = 182) and missing data or duplicate (*n* = 37), a remaining 472 patient were screened for inclusion. Three hundred and fifty-seven infants with GPB sepsis were finally included in the study, equal to an incidence of 1.47/1000 LB, of which 65 were EOS (18.2%) and 292 were LOS (81.8%) (Table 1). Infants born at the South General Hospital (*n* = 71,999) were excluded for the incidence calculations. The general characteristics of the study population are showcased in Table 2.

**Table 1:** General characteristics of the study population

Characteristics	No (%)	EOS (No tot)	LOS (No tot)	No tot
**Gestation week**		(65)	(292)	357
Extremely pre-term (GW 22–27)	216 (60.5)	8	208	
Very pre-term (GW 28–32)	74 (20.8)	10	64	
Late pre-term (GW 33–36)	20 (5,6)	8	12	
Full-term (GW 37–42)	47 (13.1)	39	8	
**Days in NICU (median)**	69.0			357
**Male sex**	217 (60.7)			357
**Birth weight**		(63)	(291)	354
Extremely low birth weight (< 1000 g)	210 (59.3)	10	200	
Very low birthweight (< 1500 g)	66 (18.6)	4	62	
Low birthweight (< 2500 g)	22 (6.2)	8	14	
Normal birthweight (≥ 2500 g)	56 (15.8)	41	15	
**APGAR-score at 5 min (median)**	7.0			332
**Vaginal delivery**	116 (33.4)	37 (64)	79 (283)	347
**Prenatal factors**				
Maternal risk of infection	108 (31.4)	26 (63)	82 (281)	344
Antenatal steroids	220 (73.6)	15 (63)	205 (236)	299
**Postnatal inventions**				
Intubation^a^	255 (73.1)	21 (64)	234 (285)	349
Surfactant treatment	213 (61.6)	13 (64)	200 (282)	346
Central catheter during NICU-stay	288 (81.6)	37 (64)	251 (290)	353
UVC	196 (58.7)	21 (64)	175 (270)	334
UAC	255 (75.9)	24 (64)	231 (272)	336
pCVC	222 (64.5)	15 (63)	206 (281)	344
**Congenital condition**				356
None	320 (89.9)	60	263	
Heart disease	13 (3.6)	3	10	
GI-anomaly	6 (1.7)	0	6	
Kidney disease/UT-anomaly	4 (1.1)	0	4	
CNS-anomaly	1 (0.3)	1	0	
Chromosomal syndrome	10 (2.8)	1	9	
Malignancy	2 (0.6)	0	2	

^a^All patients that were given surfactant are included in the group, regardless of later continuous intubation. *GW* gestation week, *NICU* neonatal intensive care unit, *UVC* umbilical venous catheter, *UAC* umbilical arterial catheter, *pCVC* peripheral central venous catheter, *GI* gastrointestinal, *UT* urinary tract, *CNS* central nervous system

**Table 2:** Incidence of Gram-positive pathogen (total numbers and per 1000 LB)

Pathogen	No (%)	Incidence sepsis	Incidence EOS (No)	Incidence LOS (No)
**All GBS**	357 (100)	1.47	0.27 (65)	1.20 (292)
CoNS	193 (53.8)	0.79	0.02 (6)	0.77 (187)
S. aureus	88 (24.9)	0.36	0.03 (8)	0.33 (80)
GBS	49 (13.7)	0.20	0.18 (43)	0.02 (6)
Other^a^	13 (3.6)	0.05	0.02 (6)	0.03 (7)
Mixed^b^	14 (3.9)	0.06	0.01 (2)	0.05 (12)

^a^*Enterococcus faecalis* (*n* = 5), *Streptococcus pneumoniae* (*n* = 2), viridans group streptococci (*n* = 3), group A streptococci (*n* = 1), group C or G streptococci (*n* = 2).

^b^Two or more GPB simultaneously; CoNS + *S. aureus* (*n* = 4), CoNS + *Enterococcus faecalis* (*n* = 3), *S. aureus* + *Enterococcus faecalis* (*n* = 3), CoNS + GBS (*n* = 2), *S. aureus* + GBS (*n* = 1) and CoNS + *S. aureus* + *Enterococcus faecalis* (*n* = 1). *CoNS* Coagulase-negative staphylococci, *GBS* Group B streptococci, *GPB* Gram-positive bacteria

### Etiology of sepsis

**Fig. 2:**
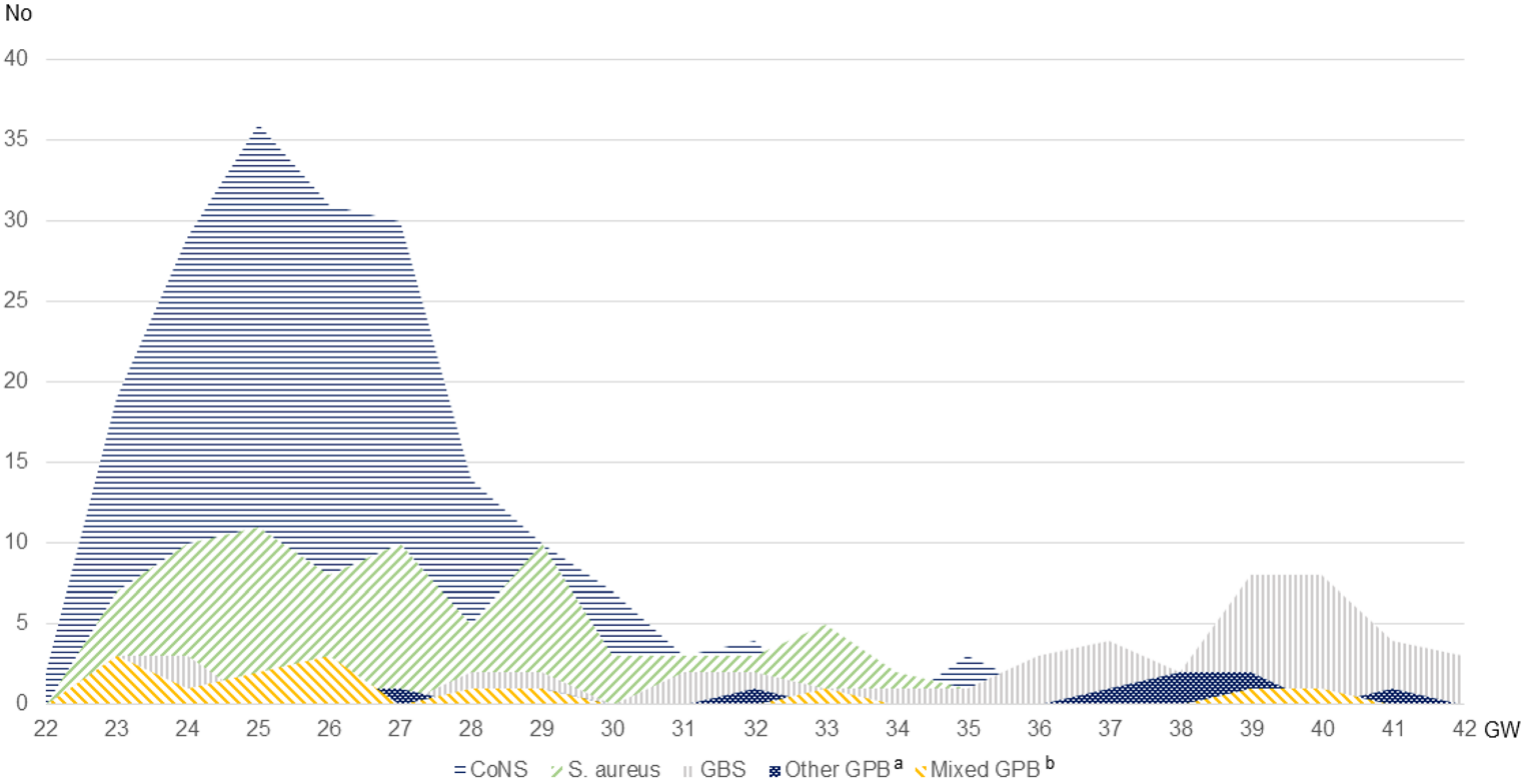
Total number of cases, according to pathogen and gestational age at birth. ^a^*Enterococcus faecalis* (*n* = 5), *Streptoococcus pneumoniae* (*n* = 2), viridans group streptococci (*n* = 3), group A streptococci (*n* = 1), group C or G streptococci (*n* = 2). ^b^Two or more GPB simultaneously; CoNS + S. aureus (*n* = 4), CoNS + *Enterococcus faecalis* (*n* = 3), S. aureus + *Enterococcus faecalis* (*n* = 3), CoNS + GBS (*n* = 2), S. aureus + GBS (*n* = 1) and CoNS + S. aureus + *Enterococcus faecalis* (*n* = 1). *EOS* early-onset sepsis, *LOS* late-onset sepsis, *CoNS* Coagulase-negative staphylococci, *GBS* Group B streptococci, *GPB* Gram-positive bacteria *, GW *gestational weeks

**Fig. 3:**
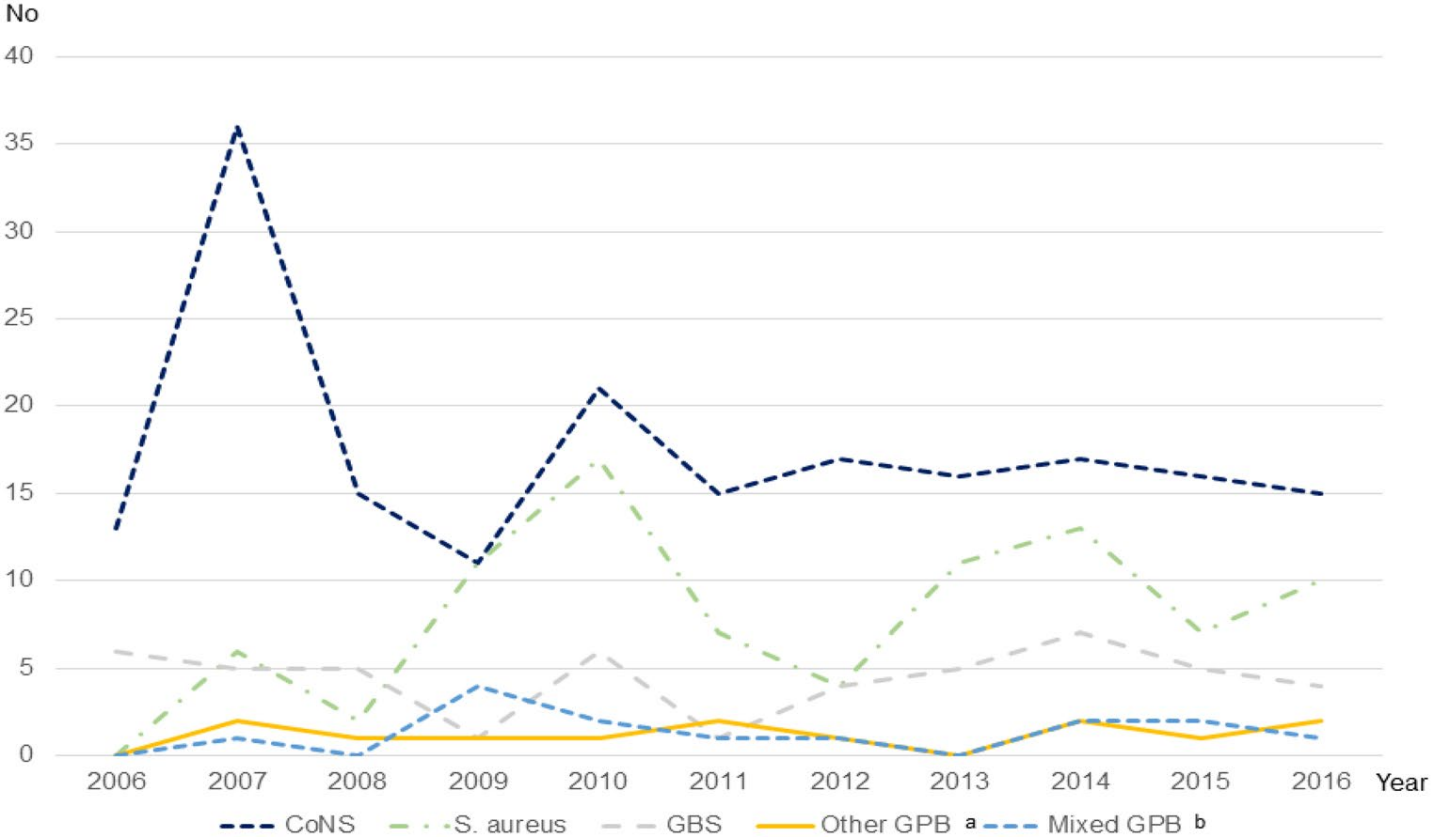
Incidence according to pathogen over the study period. ^a^*Enterococcus faecalis* (*n* = 5), *Streptococcus pneumoniae* (*n* = 2), viridans group streptococci (*n* = 3), group A streptococci (*n* = 1), group C or G streptococci (*n* = 2). ^b^Two or more GPB simultaneously; CoNS + S. aureus (*n* = 4), CoNS + *Enterococcus faecalis* (*n* = 3), S. aureus + *Enterococcus faecalis* (*n* = 3), CoNS + GBS (*n* = 2), S. aureus + GBS (*n* = 1) and CoNS + S. aureus + *Enterococcus faecalis* (*n* = 1). *EOS* early-onset sepsis, *LOS* late-onset sepsis, *CoNS* Coagulase-negative staphylococci, *GBS* Group B streptococci, *GPB* Gram-positive bacteria

**Fig. 4:**
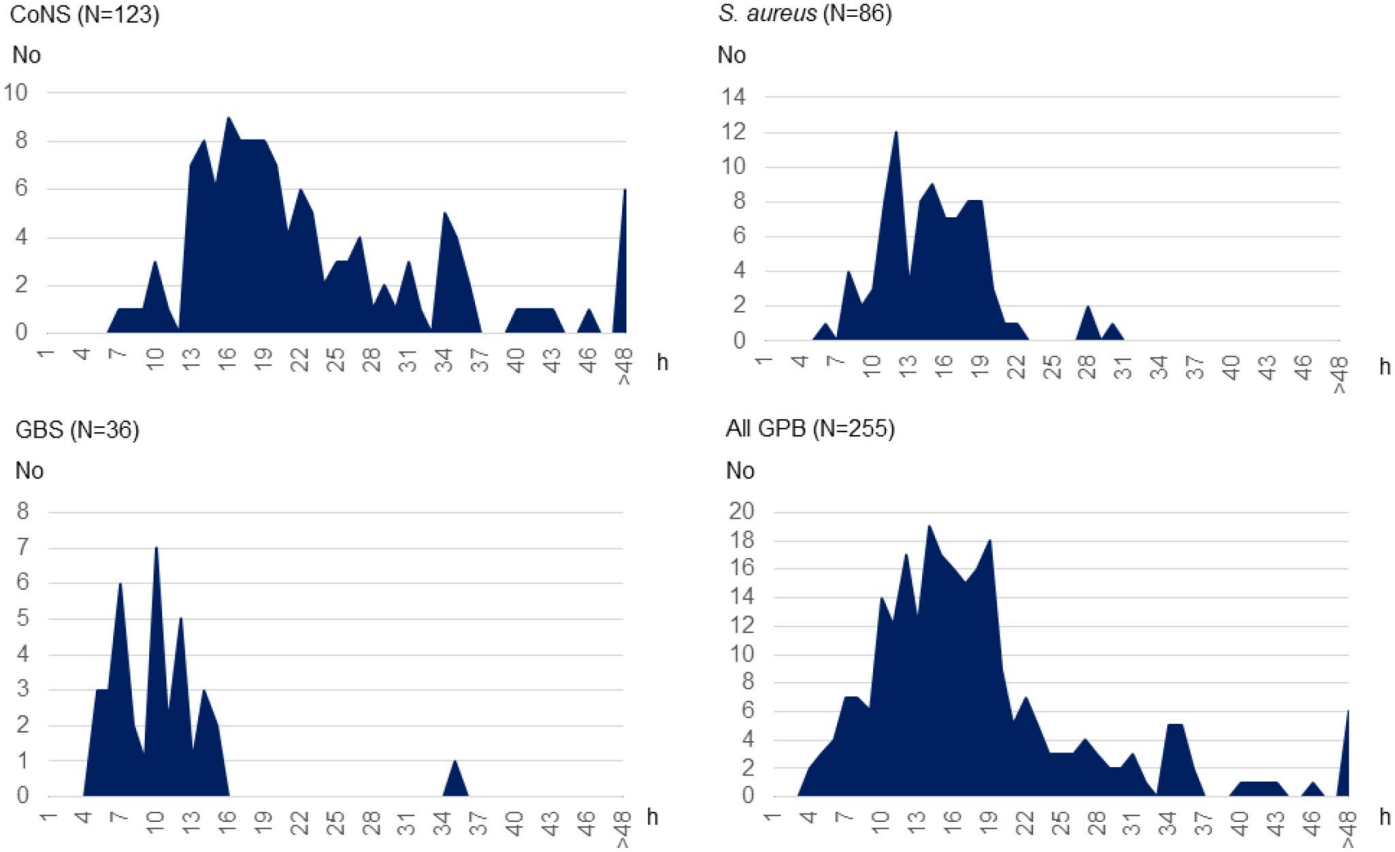
Time to bacterial growth in blood or cerebrospinal fluid (CSF) culture for different pathogens. *CoNS* Coagulase-negative staphylococci, *GBS* Group B streptococci, *GPB* Gram-positive bacteria

The most common pathogen was CoNS (*n* = 193, 53.8%), followed by *S. aureus* (*n* = 88, 24.9%) and GBS (*n* = 49, 13.7%). Figure 2 showcases an overlap in the distribution of pathogen in accordance with gestational age at birth, with a dominance of CoNS sepsis in the extremely (< 28 gestational weeks (GW) (*n* = 147, 69.0%) and very preterm born (< 32 GW) (*n* = 38, 51.6%) infants changing to *S. aureus* and GBS being the most common pathogens in the moderately preterm born (32 − 26 GW) (*n* = 6, 30.0%) and GBS in the full-term (*n* = 29, 61.7%) groups. Further, the number of *S. aureus* sepsis increased, while the number of infections from other GPB remained steady over the study period (Fig. 3). Microbiological data showed that time to growth differed depending on pathogen. Most cultures were positive within the first 24 h (*n* = 214, 83.9%), but almost 1/3 of CoNS cultures were positive only later (*n* = 40, 32.5%) (Fig. 4).

### Morbidity and mortality

The sepsis fatality rate (SFR) was 2.8% (*n* = 10), whereof 0% (*n* = 0) for EOS and 3.4% (*n* = 10) for LOS (Table 3). Of the SFR cases, 9/10 were born extremely premature; GW 22 (*n* = 1), GW 23 (*n* = 5), GW 24 (*n* = 2) and GW 26 (*n* = 1). One infant the SFR group was born in GW 35. The total case fatality rate (CFR) was 9.5% (*n* = 34), whereof 0.3% (*n* = 1) was EOS and 97.1% (*n* = 33) LOS. Infections with two or more GPB at the same time had a high SFR (*n* = 2, 14.3%) and CFR (*n* = 4, 28.6%). There were no mortality cases of GBS sepsis in this study, despite 10.2% of the GBS infections developed meningitis (*n* = 5), whereof four had seizures and one had multiple abscesses in the brain. The median age at death was 46 days. Of all neonates with GPB sepsis, 200 had an adverse outcome during their stay at NICU (56.0%) (Table 3). Forty infants had a GPB sepsis leading to septic shock (11.3%).

**Table 3:** Outcome of GPB-sepsis

Outcome	No (%)	EOS	LOS	SFR^a^ (%)	SFR^a^ EOS (%)	SFR^a^ LOS (%)	CFR^b^ (%)	CFR^b^ EOS (%)	CFR^b^ LOS (%)	No tot
**Mortality all GBS**	357 (100)	65	292	10 (2.8)	0 (0)	10 (3.4)	34 (9.5)	1 (0.3)	33 (11.3)	**357**
CoNS	193 (54.0)	5	188	6 (3.1)	0 (0)	6 (3.2)	22 (11.4)	0 (0)	22 (11.7)	193
S. aureus	88 (24.6)	9	79	1 (1.1)	0 (0)	1 (1.3)	5 (5.7)	1 (11.1)	5 (6.3)	88
GBS	49 (13.7)	43	6	0 (0)	0 (0)	0 (0)	0 (0)	0 (0)	0 (0)	49
Other^c^	13 (3.6)	6	7	1 (7.7)	0 (0)	1 (14.3)	3 (23.1)	0 (0)	3 (42.9)	13
Mixed^e^	14 (3.9)	2	12	2 (14.3)	0 (0)	2 (16.7)	4 (28.6)	0 (0)	4 (33.3)	14
**Morbidity**										
All GBS	200 (56.0)									**357**
NEC	84 (23.5)									357
- Medically treated	69 (19.3)	0	69							
- Surgically treated	15 (4.2)	0	15							
IVH 3–4 discharge	34 (10.3)	5	29							330
ROP 3–4 discharge	46 (18.6)	4	42							247
BPD	144 (50.9)	8	136							283
Septic chock	40 (11.3)	8	32							354

^a^Death within 5 days after GPB-positive culture

^b^Death before discharge from the neonatal unit

^c^*Enterococcus faecalis* (*n* = 5), *Streptococcus pneumoniae* (*n* = 2), viridans group streptococci (*n* = 3), Group A streptococci (*n* = 1), Group C/G streptococci (*n* = 2). Two or more GPB simultaneously; CoNS + S. aureus (*n* = 4), CoNS + *Enterococcus faecalis* (*n* = 3), S. aureus + *Enterococcus faecalis* (*n* = 3), CoNS + GBS (*n* = 2), S. aureus + GBS (*n* = 1) and CoNS + S. aureus + *Enterococcus faecalis* (*n* = 1). *NEC* necrotizing enterocolitis, *IVH* intraventricular haemorrhage, *ROP* retinopathy of the newborn, *BPD* bronchopulmonary dysplasia, *CoNS* Coagulase-negative staphylococci and *GBS* Group B streptococci

### Antimicrobial resistance patterns and biomarkers

The percentage of AMR in the GPB isolates in the study material was notably high (*n* = 205, 60.0%). This high percentage was primarily driven by the elevated AMR-prevalence observed in CoNS where 95.3% (*n* = 184) of isolates demonstrated AMR as indicated in Table 4. Most CoNS isolates showed resistance to both isoxazolyl penicillin (*n* = 177, 91.7%) and gentamicin (*n* = 172, 89.1%) and more than half were displaying resistance to fusidic acid (*n* = 99, 51.3%). *S. aureus* had the lowest percentage of AMR, with only 8% AMR (*n* = 7). Of these 5/7 were resistant to clindamycin. However, only mirroring 5.7% of all cultures positive for *S. aureus* in the study. There was no methicillin-resistant *S. aureus* (MRSA) or isolates with resistance to vancomycin. An increased concentration of C-reactive protein (CRP) in response to GPB sepsis was observed in most patients (*n* = 312, 90.2%) (Supplementary Table S1). However, 34 patients did not have an elevated CRP, despite an ongoing GPB sepsis (9.8%), of which most were caused by CoNS (*n* = 28) in infants born pre-term. In the group extremely premature neonates (Table 3). One hundred and forty out of 350 (40.0%) infants had a platelet (PLT) count < 100 × 10^9^/l related to GPB sepsis (Supplementary Figure S1 and S2).

**Table 4:** Antimicrobial resistance

Pathogen and antibiotic	No AMR (%)	No total
**All GPB**	205 (60.0)	342
** CoNS**	184 (95.3)	193
Penicillin		
Isoxazolylpenicillin	177 (91.7)	
Aminoglycosides		
Gentamicin	172 (89.1)	
Amikacin	27 (14.0)	
Glycopeptides		
Teicoplanin	17 (8.8)	
Lincosamines		
Clindamycin	75 (38.9)	
Oxazolidinones		
Linezolid	1 (0.5)	
Others		
Fusidic acid	99 (51.3)	
Trimethoprim/sulfamethoxazole	92 (47.7)	
Rifampicin	8 (4.1)	
** S. aureus**	7 (8.0)	88
Penicillin		
Isoxazolylpenicillin	0 (0)	
Lincosamines		
Clindamycin	5 (5.7)	
Others		
Fusidic acid	2 (2.3)	
** GBS**	11 (22.4)	49
Macrolides and lincosamines		
Clindamycin	3 (6.1)	
Erythromycin	3 (6.1)	
Others		
Tetracycline	2 (4.1)	
** Other GPB** ^a^	4 (25.0)	13
Aminoglycosides		
Gentamicin	2 (12.5)	
Lincosamines		
Clindamycin	1 (6.3)	

^a^*Enterococcus faecalis* (*n* = 5), *Streptoococcus pneumoniae* (*n* = 2), viridans group streptococci (*n* = 3), group A streptococci (*n* = 1), group C or G streptococci (*n* = 2). *AMR* Antimicrobial resistance, *CoNS* Coagulase-negative staphylococci, *GBS* Group B streptococci, *GPB* Gram-positive bacteria and *No* Number

## Discussion

Neonatal sepsis is a feared condition, at the same time as consensus for diagnostics criteria are missing, the potential negative effects of unnecessary antibiotic treatment plentiful, and AMR is an upcoming crisis [2, 8, 16]. A cornerstone in a factful approach to AMR is knowledge of current spectrum of bacterial pathogens, as well as their AMR patterns. In this study, we have described incidence, morbidity, mortality, AMR patterns, and biomarkers of GPB sepsis in neonates to increase the knowledgebase for clinicians to use in diagnostics and treatment of neonatal sepsis.

The incidence of GPB sepsis in this study was 1.47/1000 LB, which is more than 4 times the incidence for GNB sepsis (0.35/1000 LB) in our area, but with a lower SFR (2.8% versus 16.8%) and CFR (9.5% versus 28.0%) [14]. This result is in line with previous studies showing a higher mortality rate for GNB sepsis compared to GPB sepsis [10, 26, 27]. Few studies have reported on GPB-sepsis separately, but our GPB-SFR in LOS is like that of a recent Norwegian study [27]. In EOS, most affected infants were born full-term and GBS was the most common pathogen, concordant to previous international and Swedish studies [1, 28–30]. However, the GBS-EOS incidence (0.18/1000 LB) in our study was lower than both earlier Swedish and international reports [28–30], but higher than a recent report from Poland [31]. Interestingly, there were no fatal cases of GBS sepsis in this study, something that contradicts an often-described mortality in neonatal GBS sepsis of 7–9% [1, 28]. This is presumably a result of an effective infection control and the Swedish risk factor based intrapartum antibiotic prophylactic treatment regimen, implemented in 2008. A decrease of GBS-EOS has been reported during the same timeframe [32].

Noticeable was that all SFR cases in this study were LOS and that 9/10 of them were born extremely preterm, the latter in line with previous findings [26]. CoNS, where most isolates came from infants born extremely- or very preterm, was the most frequent causing pathogen in LOS. This is consistent with previous studies [10, 26, 27]. The CFR for CoNS seen in our study was in line with previous studies [10, 33]. CoNS are commonly presented as a group, despite the individual bacteria’s varying virulence [3]. A distinction of individual CoNS could be of interest for future studies, to see any potential difference in outcome in neonatal sepsis caused by different bacteria within this group. It is important to consider the vulnerability of this extremely preterm born patient group, many with more than one serious condition, which could explain that we found a SFR significantly lower than the CFR for CoNS (3.1% vs. 11.4%).

Further, it is thought-provoking that we found an increasing trend in *S. aureus* sepsis over the study period (Fig. 3), similar to findings from the USA (neonatal) [34] and Europe (adults and children) [35], but in contrast to finding in Australia (neonatal) [36], Korea (neonatal) [26] and a recent international systematic review in adult patients [37]. The stable CoNS incidence indicates that the increasing numbers of *S. aureus* infections is unlikely to be related to breaches in infection control measures.

None of the *S. aureus* isolates in our study were MRSA in contrast to results from studies in the USA (28%) [38], Australia (26%) [36] and a study covering several low-and middle income countries (61%) [39]. However, an increase of MRSA-positive isolates (all sample types) has been seen in our region over the study period, according to the Public Health Institute of Sweden [40], though mostly from asymptomatic colonization of the bacteria. Besides, a high percentage of CoNS in this study were AMR (95.3%), which highlights the groups tendency to acquire AMR genes. Therefore, the risk of both current AMR as well as the risk of causing an increase in the antibiotic resistant gene-load in infants’ intestinal microbiota, should be taken into consideration when treating CoNS. For instance, 14% of the CoNS isolates were resistant to amikacin, a first line antibiotic commonly used in combination with cephalosporins or cloxacillin in LOS of unknown cause in our setting. Still, none of the CoNS isolates showed resistance to vancomycin, something that has been described from several countries [41, 42], which establishes vancomycin as first line treatment for culture-verified CoNS sepsis in neonates in our area. However, it is important to emphasize the limitation of vancomycin use to confirmed CoNS sepsis, to avoid contributing to the further development of AMR. Further, a recent Norwegian study found a wide variation in amounts of prescribed vancomycin in between sites, with no clear relation to the sepsis related mortality rates [43], which emphasis the need to use vancomycin restrictively. In addition, the WHO has classified vancomycin as “watch” in the AwaRe category. This classification indicates that vancomycin belongs to a group of antibiotics that should be used only for specific infections [16].

As noted, diagnostics of neonatal sepsis is challenging. Biomarkers are helpful, but our study showed that 9.8% of all GPB sepsis had no elevated CRP concentration, specifically apparent for infants in the lower GWs. This, together with a well described delayed increase in CRP-levels at the occurrence of an infection, makes CRP as a biomarker unreliable for early detection of neonatal sepsis [44]. It has been suggested that a combination of CRP and procalcitonin (PCT) testing could improve the diagnostic sensitivity as PCT-levels increase after just 6-hours [44, 45]. However, neither an increased CRP- nor an elevated PCT-level are specific for infections, as they can both be elevated by non-infectious factors as well [45]. Most infants with GPB born before GW 32 had a lowered PLT count (< 100). The variation in virulence levels among GPB, including CoNS, presents a complex scenario. Despite being GPB, CoNS exhibit properties that results in comparatively less inflammatory response than other GPB (Supplementary Figure S1 and S2). This distinction can be challenging to attribute solely to host factors, such as the immaturity of the host´s immune system in premature infants.

The retrospective design of this study is a limitation, due to the lack of possibility to control missing data or to re-test cultures for accuracy. Further, since we have not accounted for infants seeking care after being discharged to home, our incidence calculations could be falsely slightly underestimated, especially regarding GBS sepsis. Moreover, the vulnerability and co-morbidity of the study population cause for several possible confounders when it comes to interpreting symptoms of sepsis. Lastly, the high SFR and CFR that we found for mixed infections are difficult to interpret due to the low numbers, the fact that one of the bacteria could be a possible contaminant, and the unusual occurrence of several bloodstream bacteria at once. The population-based design and the availability to extract data from several different systems (patient records, the Swedish Neonatal Quality Register (SNQ), the microbiological laboratory at Karolinska University Hospital and from the Public Health Agency of Sweden) is a strength as it is less likely that we have missed culture positive GPB sepsis cases.

## Conclusion

Neonatal GPB sepsis is four times more prevalent than GNB sepsis in the Stockholm region. Though the mortality is only a third of that for GNB sepsis, 1/10 patients with GPB sepsis still experience fatal outcomes, and of these all were LOS and 9/10 born extremely preterm. Moreover, a significant number of patients suffer from various sequelae. In EOS and infants born full-term, GBS was the most common causative agent, but with no fatal outcomes. CoNS was the predominant pathogen in LOS and among infants born before gestational week 32. CoNS also featured the highest percentage of AMR, which should be considered when determining treatment strategies. Lastly, the increasing trend of *S. aureus* in our area indicates a need to understand underlaying reasons.

### Electronic supplementary material

Below is the link to the electronic supplementary material.

Supplementary Material 1

## Data Availability

The datasets generated during and/or analysed during the current study are not publicly available due to data protection regulations but are available from the corresponding author on reasonable request.
