# Supplementary material for: A decade of neonatal sepsis in Stockholm, Sweden: Gram-positive pathogens were four times as common as Gram-negatives
Source: Eur J Clin Microbiol Infect Dis. 2024 Mar 22;43(5):959–68. doi: 10.1007/s10096-024-04809-8 (PMC11108929; doi:10.1007/s10096-024-04809-8)
Supplement: Supplementary file 1 — Supplementary Material 1 [file 10096_2024_4809_MOESM1_ESM.docx]

**Supplementary Table S1. Biomarkers in GPB-sepsis**

| ***Gestation week*** | ***CRP<5***  ***No (%)*** | ***CRP>5***  ***No (%)*** | ***No tot*** | ***PLT<100***  ***No (%)*** | ***PLT normal value***  ***No (%)*** | ***PLT >300***  ***No (%)*** | ***No tot*** |
| --- | --- | --- | --- | --- | --- | --- | --- |
| ***All GPB*** | 34 (9.8) | 312 (90.2) | **346** | 140 (40.0) | 121 (34.6) | 119 (34.0) | **350** |
| *Extreme pre-term (GW 22–27)* | 28 (13.3) | 182 (87.1) | 210 | 97 (46.0) | 64 (30.3) | 50 (23.7) | 211 |
| *Very pre-term*  *(GW 28–32)* | 5 (6.8) | 66 (93.0) | 71 | 26 (36.1) | 30 (41.7) | 16 (22.2) | 72 |
| *Late pre-term*  *(GW 33–36)* | 0 (0) | 19 (100) | 19 | 6 (30.0) | 4 (20.0) | 10 (50.0) | 20 |
| *Full-term*  *(GW 37–42)* | 1 (2.1) | 45 (97.8) | 46 | 11 (23.4) | 23 (49.0) | 13 (27.7) | 47 |

*No* Number, *GPB* Gram-positive bacteria, PLT unit; 10^9^/l, *GW* gestation week, *PLT* platelets and *CRP* C-reactive protein.

**Supplementary Fig. S1: Maximum PLT count in accordance with pathogen**


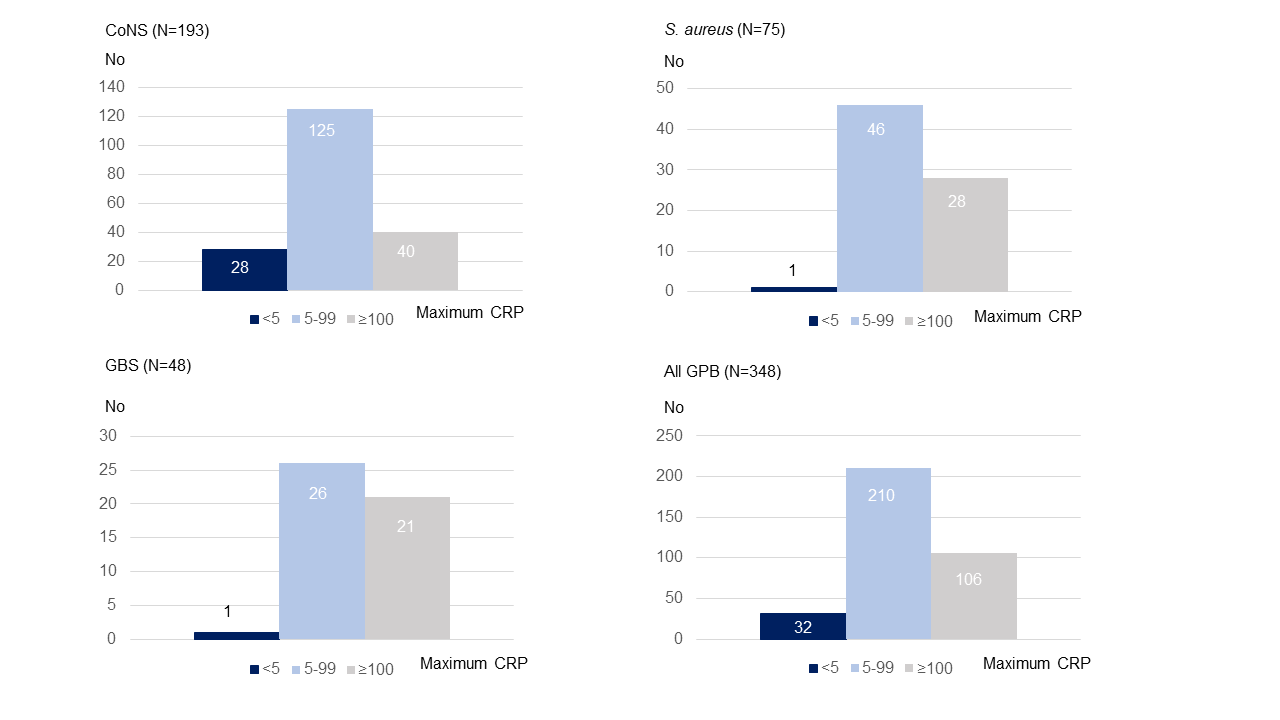


*CoNS* Coagulase-negative staphylococci, *GBS* Group B streptococci, *GPB* Gram-positive bacteria and *CRP* C-reactive protein, *PLT* platelet

**Supplementary Fig. S2: Maximum PLT count in accordance to pathogen**


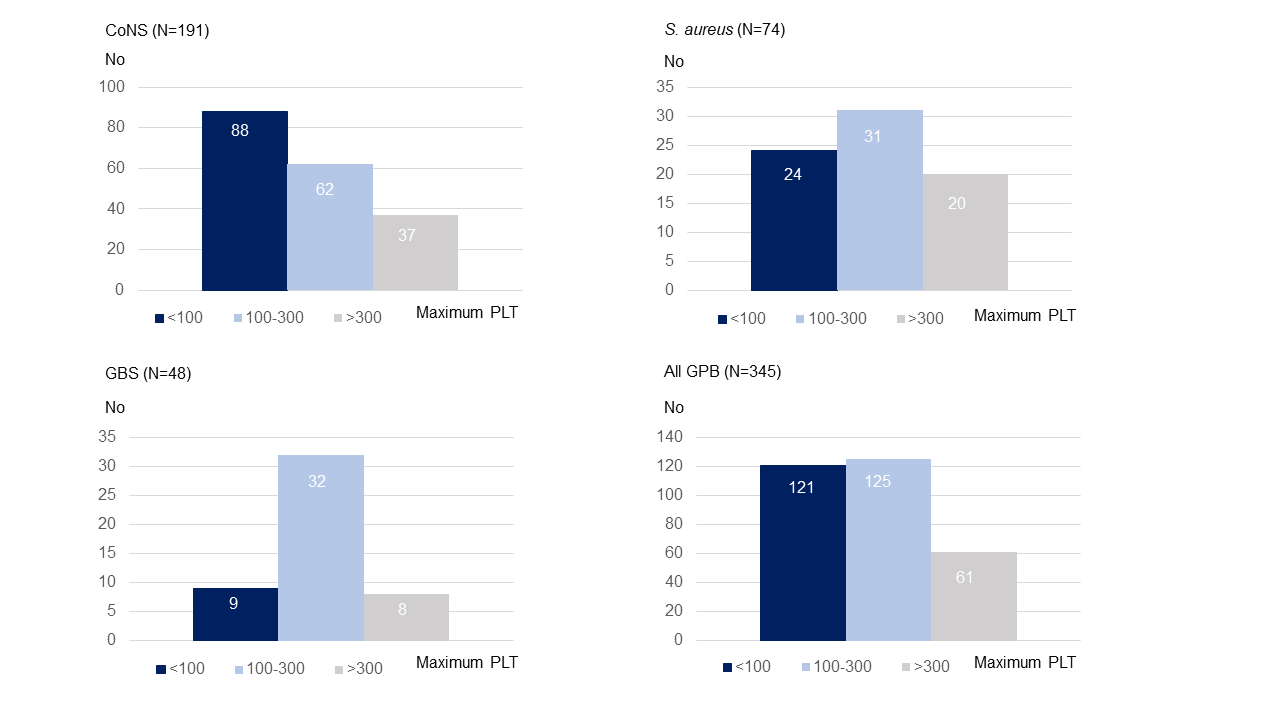


*CoNS* Coagulase-negative staphylococci, *GBS* Group B streptococci, *GPB* Gram-positive bacteria and *PLT* platelet.
